# Association of white blood cell count with breast cancer burden varies according to menopausal status, body mass index, and hormone receptor status: a case-control study

**DOI:** 10.1038/s41598-019-42234-6

**Published:** 2019-04-08

**Authors:** Byoungjin Park, Hye Sun Lee, Ji Won Lee, Seho Park

**Affiliations:** 1 Department of Family Medicine, Yonsei University College of Medicine, Seoul, 03722 Republic of Korea; 2 Biostatistics Collaboration Unit, Department of Research Affairs, Yonsei University College of Medicine, Seoul, 03722 Republic of Korea; 3 Division of Breast Surgery, Department of Surgery, Yonsei University College of Medicine, Seoul, 03722 Republic of Korea

## Abstract

Breast cancer is a heterogeneous disease that among Korean women has a peak incidence in the perimenopausal period. The full epidemiological characteristics of breast cancer in Korean women are not yet properly understood. We investigated whether white blood cell (WBC) is related to breast cancer burden according to estrogen receptor (ER) and progesterone receptor (PR) status in the context of body mass index and menopausal status. We conducted a large case-control study and compared WBC counts between patients with breast cancer (N = 4,402) and propensity score-matched controls (N = 4,402) selected from the Korean National Health and Nutrition Examination Survey (KNHANES). We stratified the study sample by ER/PR status, menopausal status, and body mass index and assessed the association between WBC count and breast cancer burden using multinomial logistic regression. Compared with controls, non-obese patients with ER^+^/PR^+^ breast cancer had significantly higher WBC counts regardless of menopausal status (OR 1.293 95% CI 1.139–1.363, *p* < 0.001 in premenopausal and OR 1.049 95% CI 1.019–1.295, *p* = 0.023 in *p*ostmenopausal). There was no relationship between WBC count and ER^+^/PR^+^ breast cancer among premenopausal obese women. Furthermore, premenopausal non-obese women and postmenopausal obese women with ER^+^/PR^+^ breast cancer had higher WBC counts than those with ER^−^/PR^−^ breast cancer. Further larger-scale prospective cohort studies are warranted to determine these associations in the future.

## Introduction

Breast cancer has by far the highest incidence of all cancer types among women around the world^1,2^. Contrary to the recent drop in the breast cancer incidence in Western countries, the incidence in Korea has been gradually rising for more than a decade^3,4^. The peak incidence of breast cancer in Korea is among women 45–49 years of age, whereas that in the USA and Canada is among women 75–79 years of age^5,6^. Although estrogen exposure, unfavorable lifestyles, and genetic factors are known to be major risk factors for breast cancer, the unique epidemiological features of breast cancer among Korean women are not properly understood. Recently, insulin resistance and metabolic syndrome were shown to be associated with an increased risk of breast cancer^7–9^. However, among Korea women, those relationships are limited to only one subtype of breast cancer in postmenopausal women^10^.

An increasing body of evidence suggests that chronic low-grade inflammation could be linked to the pathogenesis of some cancers^11–13^. White blood cell (WBC) count, an inflammatory biomarker, has become a useful predictor of certain diseases as well as a marker of infection^14–17^. An elevated WBC count, even within the normal range, has been associated with cancer incidence and mortality and with atherosclerotic cardiovascular diseases^18–20^. The role of WBC count as a surrogate for inflammation has not been examined in the context of well-known effect modifiers for breast cancer development.

Previous epidemiological studies have demonstrated that obesity, as indicated by body mass index (BMI), can influence the breast cancer risk, which can be altered differently according to menopausal status^21–23^. Several studies have attempted to identify the association between WBC counts and breast cancer risk, but no consistent evidence has been found, and those studies were not conducted without stratification by menopausal status and BMI^24,25^. Furthermore, hormone receptor positive- and negative- breast tumors are heterogeneous with respect to risk factors and etiology^26,27^. In this regard, we would like to shed light on the interaction between inflammation, as indicated by WBC count, and breast cancer burden according to estrogen receptor (ER) and progesterone receptor (PR) status in the context of body mass index and menopausal status. We conducted a case-control study to investigate whether WBC count is related to breast cancer burden according to ER/PR status in the context of body mass index and menopausal status.

## Materials and Methods

### Study population

**Figure 1:**
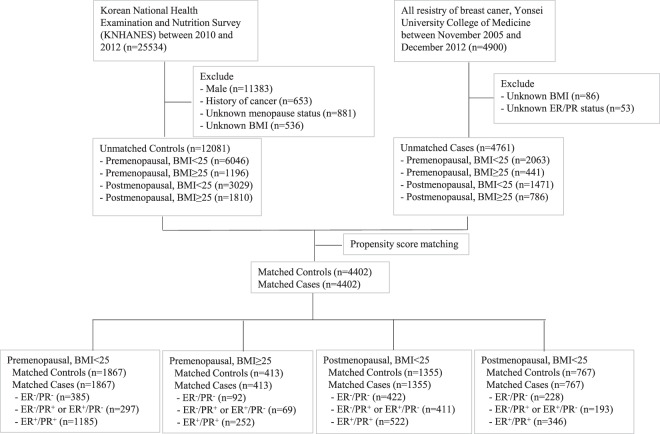
Selection of patients and controls for the study.

We selected patients with breast cancer from the breast cancer registry of the Department of Surgery, Severance Hospital, Yonsei University College of Medicine, Seoul, Republic of Korea, which includes anthropometric measures, laboratory data, personal medical history, and clinicopathological features of breast cancers. The Institutional Review Board of Yonsei University College of Medicine approved the study. We included in the study 4402 female Korean patients who were diagnosed with *in situ* or invasive carcinoma of the breast between November 2005 and December 2012 and subsequently underwent surgery for breast cancer. Throughout this period, baseline characteristics of breast cancer patients were similar and sequentially registered enough to secure proper stratification by BMI, menopausal status, and hormone receptor status. We excluded patients for whom there was no available information about menopause or BMI, those with Stage IV breast cancer, and those less than 20 years of age at the time of diagnosis (Fig. 1).

We selected 4402 propensity score-matched controls from the 2010–2012 Korean National Health and Nutrition Examination Survey (KNHANES), which was, as a fifth period survey, sampled without duplication of participants and performed on well-refined setting, while the earlier KNHANES were sampled again at every period due to the possibility of collective and relative duplication of missing data. We used households as the sampling units and selected individuals using a stratified, multistage, probability-sampling design according to geographic area, sex, and age. We excluded males, individuals with unknown menopause status or BMI, and individuals with a history of cancer. We assigned each eligible individual a sampling weight indicating the probability of being sampled. Therefore, we consider our results to appropriately represent the entire Korean population.

### Data collection

We adopted the same variables for analysis from the Severance Hospital registry and the KNHANES. Body weight and height were measured to the nearest 0.1 kg and 0.1 cm, respectively, with the participants wearing light indoor clothing without shoes. BMI was calculated as the ratio of weight (kg) to squared height (m^2^). After a 12 h overnight fast, blood samples were obtained through an antecubital vein from the controls. WBC counts were quantified using XE-2100D (Sysmex, Japan). Fasting plasma glucose, total cholesterol, triglyceride, HDL-cholesterol, aspartate aminotransferase (AST), and alanine aminotransferase (ALT) were measured using an automated chemistry analyzer (Hitachi 7600, Tokyo, Japan). Also, an overnight-fasting venous blood specimen was collected from all patients within one week after breast cancer diagnosis. WBC counts were quantified by an automated blood cell counter (ADVIA 120, NY, USA). Fasting plasma glucose, total cholesterol, triglyceride, HDL-cholesterol, AST, and ALT were measured using Hitachi 7600–110 Chemistry System Autoanalyzer (Hitachi, Tokyo, Japan). Previous study comparing automated blood cell counters has shown that there is no difference in reference intervals for WBC counts between two analyzers used in controls and cases, respectively^28^. After definitive surgery for breast cancer, tumor specimens were stained for ER and PR. Specimens with more than 1% nuclear-stained cells were defined as positive for ER and/or PR according to the guidelines of the American Society of Clinical Oncology-College of American Pathologists^29^. Menopause status, menarchial age, menopausal age, and history of breastfeeding were documented among the patients and the controls by a self-administered questionnaire. Menopause was defined as the cessation of menstruation for more than 12 months or surgical menopausal status in cases and controls.

### Statistical analysis

We compared the characteristics of the patients and propensity score-matched controls using paired t-tests for continuous variables and McNemar’s test for categorical variables. Propensity score was created using age, the most well-known confounding variable. We examined potential effects on WBC counts of obesity (BMI ≥ 25 kg/m²) and ER/PR status (ER^−^/PR^−^, ER^+^/PR^−^ or ER^−^/PR^+^, or ER^+^/PR^+^) according to menopausal status. This stratification was based on previous meta-analysis showing the different association between body weight and breast cancer risk among ER^−^/PR^−^, ER^+^/PR^−^ or ER^−^/PR^+^, or ER^+^/PR^+^ by menopausal status^27^. We performed an analysis of covariance (ANCOVA) adjusted for WBC counts of controls to compare differences in WBC counts between matched patients and controls among groups in which the patients were ER^−^/PR^−^, ER^+^/PR^−^ or ER^−^/PR^+^, or ER^+^/PR^+^. Using multinomial logistic regression, we measured the strength of correlation between WBC count (×10^3^ cells/μL) and breast cancer burden according to ER/PR status stratified by BMI and menopause after adjusting for continuous variables (age, systolic blood pressure, fasting plasma glucose, total cholesterol, triglyceride, HDL-cholesterol, ALT, and age at menarche or age at menopause) and categorical variables (breastfeeding, hypertension medication, and diabetes medication), including significant variables (*p* < 0.05) in univariate analysis with clinically important variables, while further considering multicollinearity. To test the combined effect of menopause status, BMI, and WBC, we tested their interactions with the interaction term for menopause status*BMI*WBC by multinomial logistic regression models for outcome. The interaction among menopause status, BMI, and WBC was tested at a significance level of 0.2. We conducted all analyses using the SAS statistical software, version 9.2 (SAS Institute Inc., Cary, NC, USA). All statistical tests were two-sided, with statistical significance determined by *p* < 0.05.

## Results

Table 1 shows the characteristics of the patients and matched controls according to menopausal status. The mean age of the premenopausal and postmenopausal women was 42.5 ± 6.1 years and 58.5 ± 7.7 years, respectively. The percentage of premenopausal women was 51.8%. Among the premenopausal women, the mean BMI of the controls (22.7 ± 3.1 kg/m^2^) was higher than that of the patients (22.5 ± 3.1 kg/m^2^; *p* < 0.001). There was no significant difference in mean BMI between the postmenopausal controls (24.2 ± 3.3 kg/m^2^) and patients (24.2 ± 3.2 kg/m^2^). Regardless of menopausal status, the patients in each group had higher WBC counts, systolic blood pressure, diastolic blood pressure, and fasting plasma glucose than the controls (*p* < 0.001 for each comparison). HDL cholesterol was lower in the premenopausal patients than in the matched controls, while there was no difference in HDL cholesterol between the postmenopausal patients and controls. Regardless of menopausal status, history of breastfeeding was more prevalent among the controls, whereas history of medication for hypertension was more prevalent among the patients. History of medication for diabetes was more prevalent among the postmenopausal patients than among the matched controls, however there was no difference in history of diabetes medication between the premenopausal patients and controls.

**Table 1:** Characteristics of breast cancer patients and age-matched controls according to menopausal status.

	Premenopausal women			Postmenopausal women		
	Controls^a^ (N = 2280)	Patients (N = 2280)	*p* value^b^	Controls^a^ (N = 2122)	Patients (N = 2122)	*p* value^b^
Age (years)	42.5 (6.1)	42.5 (6.1)	0.999	58.5 (7.7)	58.5 (7.7)	0.999
Body mass index (kg/m²)	22.7 (3.1)	22.5 (3.1)	<0.001	24.2 (3.3)	24.2 (3.2)	0.832
Systolic blood pressure (mmHg)	110.6 (14.4)	119.7 (13.3)	<0.001	124.1 (18.0)	128.4 (14.9)	<0.001
Diastolic blood pressure (mmHg)	72.7 (9.6)	76.7 (9.9)	<0.001	76.6 (10.0)	79.0 (9.6)	<0.001
Fasting plasma glucose (mg/dL)	92.5 (18.9)	97.3 (15.3)	<0.001	98.6 (21.9)	105.4 (25.1)	<0.001
Total cholesterol (mg/dL)	186.1 (32.7)	179.6 (31.8)	<0.001	204.0 (36.4)	197.0 (35.7)	<0.001
Triglyceride (mg/dL)	99.2 (81.4)	98.3 (67.1)	0.726	133.1 (80.8)	127.8 (71.0)	0.044
HDL cholesterol (mg/dL)	57.1 (12.5)	55.4 (12.0)	<0.001	54.2 (13.1)	53.1 (12.0)	0.020
White blood cells (×10^3^ cells/μL)	5.6 (1.6)	6.0 (1.7)	<0.001	5.7 (1.6)	5.9 (1.6)	<0.001
Aspartate aminotransferase (IU)	18.5 (6.8)	18.9 (15.9)	0.356	23.1 (10.0)	22.9 (16.1)	0.748
Alanine aminotransferase (IU)	15.7 (11.2)	17.8 (24.6)	0.011	21.2 (17.0)	22.4 (16.3)	0.118
Age at menarche (years)	14.2 (1.7)	14.3 (1.5)	0.052	15.8 (2.0)	15.7 (1.9)	0.185
Age at menopause (years)				49.0 (4.8)	49.4 (5.4)	0.002
Breastfeeding (%)	68.5	50.9	<0.001	86.9	74.4	<0.001
Hypertension medication (%)	4.1	6.2	<0.001	30.8	37.6	<0.001
Diabetes medication (%)	1.4	1.5	0.700	8.6	13.2	<0.001

Data are expressed as the mean (standard deviation) for continuous variables or as the percentage for categorical variables. ^a^Controls are propensity score-matched data. ^b^*p* values calculated using paired t-test or McNemar’s test.

**Figure 2:**
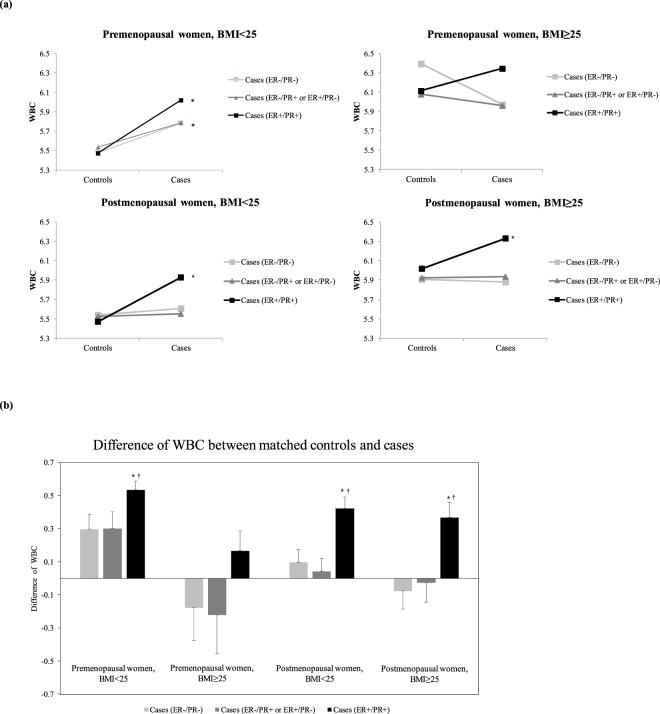
Comparison of WBC counts between patients and matched controls (ER^−^/PR^−^, ER^+^/PR^−^ or ER^−^/PR^+^, ER^+^/PR^+^) according to menopause status and obesity status. ^*^P < 0.05, calculated by paired t-test (**a**). Differences in WBC counts between patients and matched controls according to menopause status and obesity status. ^*^Difference between ER^−^/PR^−^ vs. ER^+^/PR^−^ or ER^−^/PR^+^ and ER^+^/PR^+^, *P* < 0.05, calculated by ANCOVA (adjusted WBC counts of controls). ^†^Difference between ER^+^/PR^−^ or ER^−^/PR^+^ vs. ER^−^/PR^−^ and ER^+^/PR^+^, *P* < 0.05, calculated by ANCOVA (adjusted WBC counts of controls) (**b**).

Premenopausal non-obese women with ER^−^/PR^−^ or ER^+^/PR^+^ breast cancer had higher WBC counts than their matched controls (*p* = 0.010 and *p* < 0.001, respectively). Postmenopausal women with ER^+^/PR^−^ or ER^−^/PR^+^ breast cancer exhibited similar trends, but the difference between the patients and controls was not significant. In contrast to the patterns in premenopausal non-obese women, premenopausal obese women with ER^−^/PR^−^ breast cancer or ER^+^/PR^−^ or ER^−^/PR^+^ breast cancer did not have higher WBC counts than their age-matched controls, and the trend for higher WBC counts relative to those in the controls was weakened in those with ER^+^/PR^+^ breast cancer (Table 2, Fig. 2a). WBC counts were significantly higher in postmenopausal women with ER^+^/PR^+^ breast cancer compared with those in matched controls, irrespective of obesity status (*p* < 0.001 and *p* = 0.014, respectively; Table 3, Fig. 2a).

**Table 2:** Characteristics of premenopausal patients with breast cancer and age-matched controls according to obesity and ER/PR status.

Non-obese	Controls^a^ (N = 385)	ER^−^/PR^−^ (N = 385)	*p* value^b^	Controls^a^ (N = 297)	ER^+^/PR^−^ or ER^−^/PR^+^ (N = 297)	*P* value^b^	Controls^a^ (N = 1185)	ER^+^/PR^+^ (N = 1185)	*p* value^b^
Age (years)	40.7 (6.7)	40.7 (6.7)	0.999	42.2 (6.4)	42.2 (6.4)	0.999	42.5 (5.8)	42.5 (5.8)	0.999
Body mass index (kg/m²)	21.3 (2.0)	21.5 (1.9)	0.123	21.8 (1.9)	21.7 (2.0)	0.668	21.7 (1.9)	21.3 (1.9)	<0.001
Systolic blood pressure (mmHg)	106.4 (12.2)	116.3 (11.8)	<0.001	108.7 (12.8)	117.7 (12.3)	<0.001	109.5 (13.5)	119.3 (13.2)	<0.001
Diastolic blood pressure (mmHg)	70.0 (8.4)	74.1 (9.2)	<0.001	71.5 (8.9)	76.0 (10.4)	<0.001	72.1 (9.5)	76.4 (9.5)	<0.001
Fasting plasma glucose (mg/dL)	90.4 (10.7)	96.8 (13.8)	<0.001	89.2 (8.3)	97.5 (16.7)	<0.001	91.3 (18.3)	95.8 (12.4)	<0.001
Total cholesterol (mg/dL)	181.4 (30.4)	175.3 (31.2)	0.028	182.7 (33.0)	179.4 (31.6)	0.427	186.0 (31.3)	178.3 (30.8)	<0.001
Triglyceride (mg/dL)	86.6 (42.6)	99.2 (70.1)	0.011	92.9 (47.9)	97.3 (75.2)	0.454	91.1 (56.1)	89.2 (54.8)	0.493
HDL cholesterol (mg/dL)	59.8 (12.8)	55.3 (12.4)	<0.001	58.1 (13.0)	55.3 (11.8)	0.015	57.7 (12.1)	56.9 (12.1)	0.238
White blood cells (×10^3^ cell/μL)	5.5 (1.4)	5.8 (1.7)	0.010	5.6 (1.5)	5.8 (1.7)	0.053	5.5 (1.6)	6.0 (1.7)	<0.001
Aspartate aminotransferase (IU)	17.5 (5.3)	20.4 (32.6)	0.210	18.0 (4.6)	18.4 (5.7)	0.558	18.1 (6.2)	18.1 (6.7)	0.970
Alanine aminotransferase (IU)	13.8 (9.6)	19.7 (50.4)	0.104	14.5 (6.2)	17.3 (10.3)	0.017	14.8 (10.5)	15.9 (10.2)	0.073
Age at menarche (years)	14.1 (1.7)	14.1 (1.6)	0.486	14.2 (1.6)	14.4 (1.6)	0.283	14.2 (1.7)	14.3 (1.5)	0.057
Breastfeeding (%)	65.1	45.9	<0.001	70.4	56.5	<0.001	67.8	48.7	<0.001
Hypertension medication (%)	1.6	3.4	0.089	5.1	5.1	0.999	2.8	5.3	0.001
Diabetes medication (%)	1.0	0.5	0.414	0.3	3.4	0.006	0.7	0.9	0.637
**Obese**	**Controls** ^**a**^ **(N = 92)**	**ER** ^**−**^ **/PR** ^**−**^ **(N = 92)**	***p*** **value** ^**b**^	**Controls** ^**a**^ **(N = 69)**	**ER** ^**+**^ **/PR** ^**−**^ **or** **ER** ^**−**^ **/PR** ^**+**^ **(N = 69)**	***P*** **value** ^**b**^	**Controls** ^**a**^ **(N = 252)**	**ER** ^**+**^ **/PR** ^**+**^ **(N = 252)**	***p*** **value** ^**b**^
Age (years)	42.1 (6.4)	42.1 (6.4)	0.999	43.8 (6.4)	43.8 (6.4)	0.999	44.9 (5.2)	44.9 (5.1)	0.999
Body mass index (kg/m²)	27.3 (1.9)	27.2 (2.1)	0.568	28.0 (2.5)	27.9 (3.1)	0.879	27.7 (2.5)	27.5 (2.9)	0.298
Systolic blood pressure (mmHg)	118.8 (18.3)	124.5 (12.8)	0.019	118.8 (15.1)	123.7 (13.7)	0.013	119.4 (16.1)	126.7 (13.9)	<0.001
Diastolic blood pressure (mmHg)	77.4 (10.1)	81.3 (10.7)	0.006	76.8 (9.8)	78.5 (9.4)	0.227	78.3 (10.0)	81.4 (9.5)	<0.001
Fasting plasma glucose (mg/dL)	102.9 (38.6)	98.9 (18.9)	0.517	105.0 (38.4)	103.6 (17.6)	0.881	99.4 (21.1)	104.1 (23.5)	0.101
Total cholesterol (mg/dL)	199.6 (50.5)	186.1 (38.8)	0.158	189.0 (36.3)	188.9 (42.4)	0.992	192.2 (33.4)	189.6 (31.3)	0.558
Triglyceride (mg/dL)	158.6 (279.0)	129.8 (133.5)	0.452	135.5 (108.2)	127.9 (64.3)	0.655	128.1 (67.3)	117.0 (55.6)	0.095
HDL cholesterol (mg/dL)	52.5 (12.2)	50.6 (9.8)	0.369	49.8 (12.6)	50.6 (11.6)	0.714	53.0 (10.8)	52.2 (11.1)	0.526
White blood cells (×10^3^ cell/μL)	6.4 (1.7)	6.0 (19)	0.108	6.1 (2.0)	6.0 (1.7)	0.731	6.1 (1.8)	6.3 (2.0)	0.201
Aspartate aminotransferase (IU)	19.8 (6.7)	20.7 (8.5)	0.548	24.5 (20.6)	22.4 (11.4)	0.672	21.0 (7.8)	20.0 (13.4)	0.473
Alanine aminotransferase (IU)	20.4 (12.2)	21.4 (15.6)	0.750	21.5 (20.5)	24.4 (12.9)	0.558	22.1 (15.1)	22.1 (20.2)	0.994
Age at menarche (years)	14.2 (1.7)	14.0 (1.7)	0.281	14.0 (1.6)	14.3 (1.6)	0.189	14.4 (1.9)	14.4 (1.6)	0.928
Breastfeeding (%)	71.1	57.8	0.064	72.1	60.3	0.144	72.8	57.6	<0.001
Hypertension medication (%)	10.9	9.8	0.818	2.9	11.6	0.033	10.7	13.1	0.386
Diabetes medication (%)	5.4	3.3	0.414	4.5	1.5	0.317	4.0	3.2	0.617

Data are expressed as the mean (standard deviation) for continuous variables or the percentage for categorical variables. Non-obese and obese were defined as body mass index <25 kg/m² and ≥25 kg/m², respectively. ^a^Controls are propensity score-matched data. ^b^*p* values calculated using paired t-test or McNemar’s test.

**Table 3:** Characteristics of postmenopausal patients with breast cancer and age-matched controls according to obesity and ER/PR status.

Non-obese	Controls^a^ (N = 422)	ER^−^/PR^−^ (N = 422)	*p* value^b^	Controls^a^ (N = 11)	ER^+^/PR^−^ or ER^−^/PR^+^ (N = 411)	*P* value^b^	Controls^a^ (N = 522)	ER^+^/PR^+^ (N = 522)	*p* value^b^
Age (years)	57.2 (6.7)	57.2 (6.7)	0.999	58.5 (7.3)	58.5 (7.3)	0.999	57.7 (8.0)	57.7 (8.0)	0.999
Body mass index (kg/m²)	22.2 (1.9)	22.2 (1.6)	0.846	22.3 (1.8)	22.3 (1.7)	0.623	22.3 (1.7)	22.4 (1.7)	0.282
Systolic blood pressure (mmHg)	120.7 (18.7)	126.1 (14.7)	<0.001	124.0 (19.0)	126.9 (15.2)	0.013	121.6 (17.6)	128.2 (15.0)	<0.001
Diastolic blood pressure (mmHg)	75.7 (9.9)	78.4 (9.4)	<0.001	76.4 (10.2)	77.0 (9.4)	0.324	75.1 (10.0)	79.2 (9.7)	<0.001
Fasting plasma glucose (mg/dL)	98.4 (22.1)	103.5 (21.7)	0.019	95.2 (12.3)	103.5 (23.6)	<0.001	95.2 (19.6)	103.3 (24.4)	<0.001
Total cholesterol (mg/dL)	202.2 (35.7)	195.2 (37.4)	0.047	200.8 (31.7)	197.8 (31.8)	0.442	200.8 (34.3)	196.6 (37.3)	0.2211
Triglyceride (mg/dL)	122.9 (76.4)	119.2 (64.6)	0.510	121.0 (77.1)	116.3 (66.5)	0.396	118.6 (74.5)	122.2 (73.3)	0.5169
HDL-cholesterol (mg/dL)	55.1 (13.6)	54.7 (12.6)	0.651	56.4 (13.6)	54.5 (12.9)	0.074	56.0 (12.8)	55.1 (11.9)	0.312
White blood cells (×10^3^ cell/μL)	5.5 (1.5)	5.6 (1.4)	0.526	5.5 (1.7)	5.6 (1.6)	0.822	5.5 (1.4)	5.9 (1.5)	<0.001
Aspartate aminotransferase (IU)	23.3 (12.0)	21.6 (10.0)	0.131	22.2 (5.6)	23.9 (23.1)	0.429	22.0 (6.8)	23.4 (21.5)	0.330
Alanine aminotransferase (IU)	21.8 (23.2)	20.6 (13.1)	0.523	18.2 (6.8)	20.7 (12.9)	0.046	18.3 (10.2)	22.0 (21.5)	0.019
Age at menarche (years)	15.7 (1.9)	15.8 (1.8)	0.512	15.6 (2.0)	15.7 (2.0)	0.909	15.8 (2.0)	15.6 (2.0)	0.195
Age at menopause (years)	49.0 (4.6)	49.4 (5.1)	0.148	49.3 (4.5)	50.0 (4.9)	0.088	48.4 (5.0)	48.5 (6.2)	0.608
Breastfeeding (%)	82.6	73.8	0.002	87.0	69.9	<0.0001	86.1	70.1	<0.001
Hypertension medication (%)	22.0	28.0	0.030	25.3	27.5	0.469	21.8	30.3	<0.001
Diabetes medication (%)	6.2	10.0	0.029	5.6	10.7	0.009	7.3	10.6	0.058
**Obese**	**Controls** ^**a**^ **(N = 228)**	**ER** ^**−**^ **/PR** ^**−**^ **(N = 228)**	***p*** **value** ^**b**^	**Controls** ^**a**^ **(N = 193)**	**ER** ^**+**^ **/PR** ^**−**^ **or** **ER** ^**−**^ **/PR** ^**+**^ **(N = 193)**	***P*** **value** ^**b**^	**Controls** ^**a**^ **(N = 346)**	**ER** ^**+**^ **/PR** ^**+**^ **(N = 346)**	***p*** **value** ^**b**^
Age (years)	59.5 (7.6)	59.5 (7.6)	0.999	59.5 (7.4)	59.5 (7.4)	0.999	60.4 (8.2)	60.4 (8.2)	0.999
Body mass index (kg/m²)	27.6 (2.3)	27.4 (2.1)	0.500	27.6 (2.7)	27.3 (2.1)	0.174	27.5 (2.7)	27.7 (2.5)	0.237
Systolic blood pressure (mmHg)	127.8 (16.5)	129.0 (14.2)	0.374	127.3 (15.8)	130.3 (14.4)	0.045	127.8 (17.2)	131.8 (14.8)	<0.001
Diastolic blood pressure (mmHg)	78.3 (9.5)	80.2 (8.7)	0.041	78.7 (9.2)	80.0 (9.8)	0.176	78.2 (10.0)	80.5 (10.0)	0.002
Fasting plasma glucose (mg/dL)	103.4 (25.1)	109.0 (27.4)	0.114	104.2 (24.6)	108.1 (30.6)	0.463	101.5 (26.2)	109.2 (26.7)	0.014
Total cholesterol (mg/dL)	203.9 (40.5)	196.2 (34.0)	0.146	215.5 (37.7)	196.7 (35.3)	0.002	209.4 (39.3)	200.2 (35.3)	0.041
Triglyceride (mg/dL)	160.2 (87.0)	143.0 (62.6)	0.044	161.8 (88.4)	129.9 (60.2)	<0.001	149.0 (79.8)	152.6 (85.3)	0.641
HDL-cholesterol (mg/dL)	51.2 (12.2)	49.8 (10.6)	0.294	51.6 (12.0)	51.5 (11.2)	0.956	50.4 (12.0)	49.2 (9.6)	0.247
White blood cells (×10^3^ cell/μL)	5.9 (1.5)	5.9 (1.6)	0.867	5.9 (1.5)	5.9 (1.5)	0.953	6.0 (1.6)	6.3 (1.6)	0.014
Aspartate aminotransferase (IU)	25.3 (15.7)	23.3 (9.3)	0.239	23.9 (8.2)	21.0 (7.0)	0.026	23.5 (9.2)	23.5 (10.8)	0.974
Alanine aminotransferase (IU)	23.7 (15.8)	24.3 (14.3)	0.740	25.0 (13.7)	21.4 (11.6)	0.064	24.1 (22.4)	25.8 (15.3)	0.399
Age at menarche (years)	16.1 (2.1)	16.0 (1.9)	0.702	16.1 (2.3)	15.7 (2.0)	0.056	15.8 (2.0)	15.7 (1.8)	0.478
Age at menopause (years)	49.1 (4.8)	50.3 (5.0)	0.006	49.7 (4.4)	50.2 (4.4)	0.281	49.1 (5.3)	49.3 (5.5)	0.686
Breastfeeding (%)	90.8	83.7	0.020	91.7	80.7	0.002	88.1	77.3	<0.001
Hypertension medication (%)	46.5	57.0	0.016	42.5	45.6	0.527	44.8	55.2	0.0058
Diabetes medication (%)	12.7	16.2	0.276	16.1	18.1	0.586	10.1	19.1	0.001

Data are expressed as the mean (standard deviation) for continuous variables or the percentage for categorical variables. Non-obese group and obese were defined as body mass index<25 kg/m² and ≥25 kg/m², respectively. ^a^Controls are propensity score-matched data. ^b^*p* values calculated using paired t-test or McNemar’s test.

Figure 2b shows the differences in WBC counts between patients and matched controls by ER/PR status, menopause status, and obesity status. ANCOVA showed that the difference in WBC counts between patients and matched controls was significantly greater for premenopausal non-obese women with ER^+^/PR^+^ breast cancer than for those with ER^−^/PR^−^ breast cancer or ER^+^/PR^−^ or ER^−^/PR^+^ breast cancer (*p* = 0.020 and *p* = 0.038, respectively). Among postmenopausal women, the difference in WBC counts between patients and controls was greater for patients with ER^+^/PR^+^ breast cancer than for those with ER^−^/PR^−^ breast cancer or ER^+^/PR^−^ or ER^−^/PR^+^ breast cancer, regardless of whether the women were obese (*p* = 0.002 and *p* < 0.001, respectively) or non-obese (*p* = 0.002 and *p* = 0.009, respectively).

**Figure 3:**
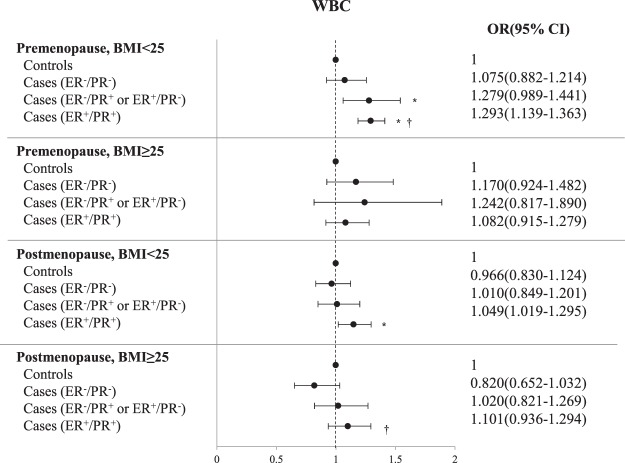
Forest plot. Models of premenopausal women were adjusted for age, systolic blood pressure, fasting plasma glucose, total cholesterol, triglyceride, HDL cholesterol, alanine aminotransferase, age at menarche, breastfeeding, hypertension medication, and diabetes medication. Models of postmenopausal women were adjusted for age, systolic blood pressure, fasting plasma glucose, total cholesterol, triglyceride, HDL cholesterol, alanine aminotransferase, age at menopause, breastfeeding, hypertension medication, and diabetes medication. ^*^Difference between control and ER^−^/PR^−^, ER^+^/PR^−^ or ER^−^/PR^+^, or ER^+^/PR^+^; *P* < 0.05, calculated by multinomial logistic regression analysis. ^†^Difference between ER^−^/PR^−^ and ER^+^/PR^−^ or ER^−^/PR^+^, or ER^+^/PR^+^; *P* < 0.05, calculated by multinomial logistic regression.

Finally, we assessed the association between WBC count and breast cancer burden according to ER/PR status, BMI, and menopausal status. For the analysis, we used multinomial logistic regression analysis after adjustment for age, systolic blood pressure, fasting plasma glucose, total cholesterol, triglyceride, HDL cholesterol, alanine aminotransferase, the use of hypertension and diabetes medications, history of breastfeeding, menarchial age, and menopausal age (Table 4, Fig. 3). Premenopausal non-obese patients with ER^+^/PR^+^ breast cancer had higher WBC counts than their age-matched controls [odds ratio (95% CI) = 1.293 (1.139–1.363), *p* < 0.001]. Likewise, postmenopausal non-obese patients with ER^+^/PR^+^ breast cancer had higher WBC counts compared to their age-matched controls [odds ratio (95% CI) = 1.049 (1.019–1.295), *p* = 0.023]. Furthermore, premenopausal non-obese patients with ER^+^/PR^+^ breast cancer had higher WBC counts than premenopausal non-obese patients with ER^−^/PR^−^ breast cancer [odds ratio (95% CI) = 1.203 (1.019–1.420), *p* = 0.029]. Similarly, postmenopausal obese patients with ER^+^/PR^+^ breast cancer had higher WBC counts than postmenopausal obese patients with ER^−^/PR^−^ breast cancer [odds ratio (95% CI) = 1.342 (1.023–1.760), *p* = 0.034]. Menopause*WBC, BMI*WBC, and menopause*BMI*WBC were all shown to have significant interactions by multinomial logistic regression models for outcome (interaction *p* value = 0.126, 0.002, and 0.184, respectively).

**Table 4:** Multinomial logistic regression analysis showing the strength of correlation between WBC count and breast cancer status.

	Premenopause, BMI < 25 kg/m²		Premenopause, BMI ≥ 25 kg/m²	
	OR (95% CI)	*p* value^a^	OR (95% CI)	*p* value^a^
Cases (ER^−^/PR^−^) vs. Controls	1.075 (0.921–1.255)	0.358	1.170 (0.924–1.482)	0.193
Cases (ER^−^/PR^+^ or ER^+^/PR^−^) vs. Controls	1.279 (1.061–1.541)	0.009	1.242 (0.817–1.890)	0.310
Cases (ER^+^/PR^+^) vs. Controls	1.293 (1.185–1.411)	<0.001	1.082 (0.915–1.279)	0.356
Cases (ER^−^/PR^+^ or ER^+^/PR^−^) vs. Cases (ER^−^/PR^−^)	1.190 (0.941–1.504)	0.146	1.062 (0.663–1.700)	0.802
Cases (ER^+^/PR^+^) vs. Cases (ER^−^/PR^−^)	1.203 (1.019–1.42)	0.028	0.925 (0.704–1.214)	0.573
Cases (ER^−^/PR^+^ or ER^+^/PR^−^) vs. Cases (ER^−^/PR^−^)	1.011 (0.834–1.226)	0.910	0.871 (0.562–1.349)	0.535
	**Postmenopause, BMI < 25 kg/m²**		**Postmenopause, BMI ≥ 25 kg/m²**	
	**OR (95% CI)**	***p*** **value** ^**b**^	**OR (95% CI)**	***p*** **value** ^**b**^
Cases (ER^−^/PR^−^) vs. Controls	0.966 (0.830–1.124)	0.653	0.820 (0.652–1.032)	0.091
Cases (ER^−^/PR^+^ or ER^+^/PR^−^) vs. Controls	1.010 (0.849–1.201)	0.912	1.020 (0.821–1.269)	0.855
Cases (ER^+^/PR^+^) vs. Controls	1.149 (1.019–1.295)	0.023	1.101 (0.936–1.294)	0.245
Cases (ER^−^/PR^+^ or ER^+^/PR^−^) vs. Cases (ER^−^/PR^−^)	1.045 (0.838–1.304)	0.694	1.244 (0.914–1.693)	0.165
Cases (ER^+^/PR^+^) vs. Cases (ER^−^/PR^−^)	1.189 (0.989–1.430)	0.064	1.342 (1.023–1.760)	0.033
Cases (ER^−^/PR^+^ or ER^+^/PR^−^) vs. Cases (ER^−^/PR^−^)	1.138 (0.930–1.392)	0.210	1.079 (0.830–1.403)	0.571

^a^Premenopausal model: Adjusted for age, systolic blood pressure, fasting plasma glucose, total cholesterol, triglyceride, HDL-cholesterol, ALT, age at menarche, breastfeeding, hypertension medication, and diabetes medication.

^b^Postmenopausal model: Adjusted for age, systolic blood pressure, fasting plasma glucose, total cholesterol, triglyceride, HDL-cholesterol, ALT, age at menopause, breastfeeding, hypertension medication, and diabetes medication.

## Discussion

In this case-control study, we found that WBC count was associated differently with breast cancer burden depending on menopausal status, BMI, and ER/PR status. Premenopausal non-obese women with ER^+^/PR^+^ breast cancer had elevated WBC counts compared with both controls and premenopausal non-obese women with ER^−^/PR^−^ breast cancer. Those trends were weaker in premenopausal obese women. Postmenopausal non-obese women with ER^+^/PR^+^ breast cancer had elevated WBC counts compared with controls, while postmenopausal obese women with ER^+^/PR^+^ breast cancer had elevated WBC counts compared with postmenopausal obese women with ER^−^/PR^−^ breast cancer.

Emerging evidence suggests that chronic low-grade inflammation plays an important role in cancer development. Both menopause and obesity can also play a crucial role in the development of breast cancer^30^, but their interaction may differ according to menopausal status^22,31,32^. Positive associations between obesity and breast cancer risk have been consistently observed in postmenopausal women^33–36^. However, there is substantial evidence that there is an inverse association between obesity and breast cancer risk in premenopausal women^37–39^. Our results showed that WBC count was not associated with breast cancer burden in premenopausal obese women.

WBCs, including neutrophils, monocytes, and eosinophils, produce reactive oxygen species (ROS) and nitric oxide species (NOS), which are chemically reactive molecules^40^. Unless ROS and NOS are properly neutralized by the antioxidant defense system, they can cause damage to cellular proteins, lipids, and DNA that may lead to the accumulation of genetic instability, affecting single nucleotide polymorphisms (SNPs) or upregulating the PI3K-Akt pathway for carcinogenesis^41^. Large-sample studies that attempted to evaluate the association between WBC counts and breast cancer risk without stratification by menopausal status and obesity have produced inconsistent results^24,25^. A prospective study demonstrated that leukocyte counts may be a predictor of breast cancer, but the study included only postmenopausal women^20^. Akinbami *et al*.^42^ reported that WBC counts were higher in patients with breast cancer than in controls, but their study did not include information about menopausal status. Okuturlar *et al*. showed that neutrophil levels were associated with the risk of breast cancer, including Stage IV breast cancer^43^. None of the previous studies included ER/PR status in their analysis. A recent meta-analysis assessed the association between the neutrophil-to-lymphocyte ratio as a biomarker using WBC subtypes and breast cancer prognosis^44^. In that study, which was performed in patients with breast cancer without control, patients with a higher neutrophil-to-lymphocyte ratio had a higher relapse and a shorter overall survival. Subgroup analysis showed that studies performed in Eastern countries had perfectly homogeneous results, whereas Western countries did not. A distinguishing feature of our study is that it attempts to elucidate a role of the interaction between WBC count and ER/PR status in the context of menopause and BMI.

The prevalence of obesity among Korean women has gradually decreased since 2001^45^, but the incidence of breast cancer has increased over the last decade^4^. The age-frequency distribution of breast cancer among Korean women is unimodal, with peak incidence at 45–49 years of age^5^. Dense breast on mammography, a potent risk factor for breast cancer, is more prevalent among Korean women, especially before menopause, than among women from Western countries^46^. Those distinctive epidemiological features warrant more investigation of the interplay between well-known risk factors such as obesity and menopause and the emerging role of inflammation in cancer development.

While metabolic syndrome and insulin resistance, as inflammatory conditions, have been noticed to be associated with breast cancer development and subsequent progression^7–9^, there are controversies, regarding discrepancies according to menopause^21–23^, as well as some limitations in Korean women. One Korean cohort study in which 23,830 Korean women 50–64 years of age were examined reported that metabolic syndrome was related to the risk of breast cancer after adjustment for age and BMI^47^. That study did not account for menopausal status, although most of the participants were likely postmenopausal, nor did it consider different breast cancer subtypes. A recent epidemiological study of postmenopausal Korean women showed that insulin resistance was independently associated only with luminal B subtype breast cancer^10^, which is included in the ER^+^/PR^+^ phenotype^48^.

ER and PR are found in about two-thirds of breast cancers, representing favorable therapeutic and prognostic factors. In terms of breast cancer pathogenesis, the risk of breast cancer development associated with weight gain, a surrogate for increasing subclinical inflammation, has been shown to be higher for ER^+^/PR^+^ breast cancer than for ER^−^/PR^−^ breast cancer^27^. Approximately 40% of ER^+^ breast cancers fail to respond to hormone therapy^49^. ER and PR status can be a precipitating factor in breast cancer development through its interplay with inflammation, which may also influence endocrine resistance. Pro-inflammatory cytokines, such as tumor necrosis factor-α (TNF-α), increase the transcriptional activity of the NF-κB and JNK pathways and may subsequently induce tumorigenesis or resistance to hormone therapy^50^. In a case-case analysis of epidemiological risk factors for breast cancer, women less than 50 years of age with ER^−^/PR^−^ tumors were more likely to be obese than those with ER^+^/PR^+^ tumors^51^. Obesity *per se* has been mainly associated with postmenopausal ER^+^/PR^+^ breast cancer^27^. Our study reported that menopausal status, BMI, and WBC showed a significant interaction by multinomial logistic regression models. Considering how previous studies have shown that obesity can be associated differently with breast cancer status, our findings suggest that among non-obese women, WBC may be related to breast cancer burden. Among Korean women, breast cancer has peak incidence in the perimenopausal period, with the number of obese women being lower than that of non-obese women^10,52^.

Our study has a few limitations. First, because our study was a case-control study, the exact cause-effect relationship between WBC count and ER/PR status according to obesity status and menopausal status remains unclear. Although it is plausible that WBC may reflect underlying inflammation and, in turn, affect breast cancer risk, higher WBC counts may result from the stress that comes after receiving a cancer diagnosis. Also, WBC counts in controls and cases were measured separately in different laboratories, with very high concordance of general blood cell counts among various automated hematology analyzers^53,54^, which may have led to differential misclassification of laboratory errors. Prospective longitudinal studies are needed to verify the effects of those interactions on the development of breast cancer, especially in Asian women. Second, some inflammatory markers such as C-reactive protein, interleukin-6, serum amyloid-A, and prostaglandin E2 were not measured at the beginning of the study. Those markers can be indicators of chronic low-grade inflammation but have not been taken into consideration when relating breast cancer to the presence of obesity. Third, cases were selected from hospital registry between 2005 and 2012, but controls were selected between 2010 and 2012 from KNHANES, albeit for proper stratification in cases and for excluding duplication of participants and minimizing missing data in controls.

In conclusion, compared with those in controls, WBC counts were significantly elevated in non-obese patients with ER^+^/PR^+^ breast cancer, irrespective of menopause. Further larger-scale prospective cohort studies are warranted to determine these associations in the future.

## References

[1] Ferlay J (2015). Cancer incidence and mortality worldwide: sources, methods and major patterns in GLOBOCAN 2012. Int J Cancer.

[2] Siegel RL, Miller KD, Jemal A (2016). Cancer statistics, 2016. CA Cancer J Clin.

[3] Jemal A (2011). Global cancer statistics. CA Cancer J Clin.

[4] Jung KW (2015). Cancer statistics in Korea: incidence, mortality, survival, and prevalence in 2012. Cancer Res Treat.

[5] Bae J-M, Lee EH, Park B, Jeong J (2015). It needs adaptation to the 2015 Korean guideline for breast cancer screening. Journal of the Korean Medical Association.

[6] Forman, D., Bray, F. & Brewster, D. Cancer incidence in five continents, vol. X. Lyon: International Agency for Research on Cancer; (2013).

[7] Agnoli C (2010). Metabolic syndrome and postmenopausal breast cancer in the ORDET cohort: a nested case-control study. Nutr Metab Cardiovasc Dis.

[8] Goodwin PJ (2009). High insulin levels in newly diagnosed breast cancer patients reflect underlying insulin resistance and are associated with components of the insulin resistance syndrome. Breast Cancer Res Treat.

[9] Verheus M (2006). Serum C-peptide levels and breast cancer risk: results from the European Prospective Investigation into Cancer and Nutrition (EPIC). Int J Cancer.

[10] Nam S (2016). Association Between Insulin Resistance and Luminal B Subtype Breast Cancer in Postmenopausal Women. Medicine (Baltimore).

[11] Coussens LM, Werb Z (2002). Inflammation and cancer. Nature.

[12] Lu H, Ouyang W, Huang C (2006). Inflammation, a key event in cancer development. Molecular cancer research.

[13] Shacter E, Weitzman SA (2002). Chronic inflammation and cancer. ONCOLOGY-WILLISTON PARK THEN HUNTINGTON-.

[14] Lee CD (2001). White blood cell count and incidence of coronary heart disease and ischemic stroke and mortality from cardiovascular disease in African-American and White men and women: atherosclerosis risk in communities study. Am J Epidemiol.

[15] Lee YJ (2010). Relationship between white blood cell count and nonalcoholic fatty liver disease. Dig Liver Dis.

[16] Nagasawa N (2004). Association of white blood cell count and clustered components of metabolic syndrome in Japanese men. Circ J.

[17] Nakanishi N, Yoshida H, Matsuo Y, Suzuki K, Tatara K (2002). White blood-cell count and the risk of impaired fasting glucose or Type II diabetes in middle-aged Japanese men. Diabetologia.

[18] Erlinger TP, Muntner P, Helzlsouer KJ (2004). WBC count and the risk of cancer mortality in a national sample of U.S. adults: results from the Second National Health and Nutrition Examination Survey mortality study. Cancer Epidemiol Biomarkers Prev.

[19] Lee YJ, Lee HR, Nam CM, Hwang UK, Jee SH (2006). White blood cell count and the risk of colon cancer. Yonsei Med J.

[20] Margolis KL, Rodabough RJ, Thomson CA, Lopez AM, McTiernan A (2007). Prospective study of leukocyte count as a predictor of incident breast, colorectal, endometrial, and lung cancer and mortality in postmenopausal women. Arch Intern Med.

[21] Anderson GL, Neuhouser ML (2012). Obesity and the risk for premenopausal and postmenopausal breast cancer. Cancer Prev Res (Phila).

[22] Rose DP, Vona-Davis L (2010). Interaction between menopausal status and obesity in affecting breast cancer risk. Maturitas.

[23] Suba Z (2013). Circulatory estrogen level protects against breast cancer in obese women. Recent Pat Anticancer Drug Discov.

[24] Allin KH, Bojesen SE, Nordestgaard BG (2016). Inflammatory biomarkers and risk of cancer in 84,000 individuals from the general population. Int J Cancer.

[25] Van Hemelrijck M (2011). Association between levels of C-reactive protein and leukocytes and cancer: three repeated measurements in the Swedish AMORIS study. Cancer Epidemiol Biomarkers Prev.

[26] Cianfrocca M, Goldstein LJ (2004). Prognostic and predictive factors in early-stage breast cancer. Oncologist.

[27] Vrieling A, Buck K, Kaaks R, Chang-Claude J (2010). Adult weight gain in relation to breast cancer risk by estrogen and progesterone receptor status: a meta-analysis. Breast Cancer Res Treat.

[28] Van den Bossche J (2002). Reference intervals for a complete blood count determined on different automated haematology analysers. Abx Pentra 120 Retic, Coulter Gen-S, Sysmex SE 9500, Abbott Cell Dyn 4000 and Bayer Advia 120..

[29] Hammond ME (2010). American Society of Clinical Oncology/College Of American Pathologists guideline recommendations for immunohistochemical testing of estrogen and progesterone receptors in breast cancer. J Clin Oncol.

[30] Hsieh CC, Trichopoulos D, Katsouyanni K, Yuasa S (1990). Age at menarche, age at menopause, height and obesity as risk factors for breast cancer: associations and interactions in an international case-control study. Int J Cancer.

[31] Franceschi S (1996). Body size indices and breast cancer risk before and after menopause. Int J Cancer.

[32] Suzuki R (2011). Body weight at age 20 years, subsequent weight change and breast cancer risk defined by estrogen and progesterone receptor status–the Japan public health center-based prospective study. Int J Cancer.

[33] White KK, Park SY, Kolonel LN, Henderson BE, Wilkens LR (2012). Body size and breast cancer risk: the Multiethnic Cohort. Int J Cancer.

[34] Ahn J (2007). Adiposity, adult weight change, and postmenopausal breast cancer risk. Arch Intern Med.

[35] Lahmann PH (2004). Body size and breast cancer risk: findings from the European Prospective Investigation into Cancer And Nutrition (EPIC). Int J Cancer.

[36] Morimoto LM (2002). Obesity, body size, and risk of postmenopausal breast cancer: the Women’s Health Initiative (United States). Cancer Causes Control.

[37] Harris HR, Willett WC, Terry KL, Michels KB (2011). Body fat distribution and risk of premenopausal breast cancer in the Nurses’ Health Study II. J Natl Cancer Inst.

[38] John EM, Sangaramoorthy M, Phipps AI, Koo J, Horn-Ross PL (2011). Adult body size, hormone receptor status, and premenopausal breast cancer risk in a multiethnic population: the San Francisco Bay Area breast cancer study. Am J Epidemiol.

[39] Ogundiran TO (2010). Case-control study of body size and breast cancer risk in Nigerian women. Am J Epidemiol.

[40] Ohshima H, Tazawa H, Sylla BS, Sawa T (2005). Prevention of human cancer by modulation of chronic inflammatory processes. Mutat Res.

[41] Okoh VO, Felty Q, Parkash J, Poppiti R, Roy D (2013). Reactive oxygen species via redox signaling to PI3K/AKT pathway contribute to the malignant growth of 4-hydroxy estradiol-transformed mammary epithelial cells. PLoS One.

[42] Akinbami A (2013). Full blood count pattern of pre-chemotherapy breast cancer patients in Lagos, Nigeria. Caspian J Intern Med.

[43] Okuturlar Y (2015). Utility of peripheral blood parameters in predicting breast cancer risk. Asian Pac J Cancer Prev.

[44] Wei B (2016). The neutrophil lymphocyte ratio is associated with breast cancer prognosis: an updated systematic review and meta-analysis. OncoTargets and therapy.

[45] Kang H-T (2014). Trends in prevalence of overweight and obesity in Korean adults, 1998–2009: the Korean National Health and Nutrition Examination Survey. Journal of epidemiology.

[46] Youn I, Choi S, Kook SH, Choi YJ (2016). Mammographic Breast Density Evaluation in Korean Women Using Fully Automated Volumetric Assessment. J Korean Med Sci.

[47] Lee JA, Yoo JE, Park HS (2017). Metabolic syndrome and incidence of breast cancer in middle-aged Korean women: a nationwide cohort study. Breast Cancer Res Treat.

[48] Dai X (2015). Breast cancer intrinsic subtype classification, clinical use and future trends. Am J Cancer Res.

[49] Ma X-J (2004). A two-gene expression ratio predicts clinical outcome in breast cancer patients treated with tamoxifen. Cancer cell.

[50] Dixon JM (2014). Endocrine Resistance in Breast Cancer. New Journal of Science.

[51] Yang XR (2011). Associations of breast cancer risk factors with tumor subtypes: a pooled analysis from the Breast Cancer Association Consortium studies. J Natl Cancer Inst.

[52] Kang, S. Y. *et al*. Basic Findings Regarding Breast Cancer in Korea in 2015: Data from a Breast Cancer Registry. **21**, 1–10 (2018). 10.4048/jbc.2018.21.1.1 PMC5880959 29628978

[53] Bruegel M (2015). Comparison of five automated hematology analyzers in a university hospital setting: Abbott Cell-Dyn Sapphire, Beckman Coulter DxH 800, Siemens Advia 2120i, Sysmex XE-5000, and Sysmex XN-2000. Clin Chem Lab Med.

[54] Meintker L, Ringwald J, Rauh M, Krause SW (2013). Comparison of automated differential blood cell counts from Abbott Sapphire, Siemens Advia 120, Beckman Coulter DxH 800, and Sysmex XE-2100 in normal and pathologic samples. Am J Clin Pathol.

